# Cross-generational plasticity in Atlantic silversides (*Menidia menidia*) under the combined effects of hypoxia and acidification

**DOI:** 10.1242/jeb.249726

**Published:** 2025-07-25

**Authors:** Christopher S. Murray, Ayanna Mays, Matthew Long, Neelakanteswar Aluru

**Affiliations:** ^1^ Biology Department, Woods Hole Oceanographic Institution, Woods Hole, MA 02543-1050, USA; ^2^ Marine Chemistry and Geochemistry Department, Woods Hole Oceanographic Institution, Woods Hole, MA 02543-1050, USA

**Keywords:** Early life history, Forage fish, Gene expression, Coastal marine systems, Multi-stressor, Parental effects, Phenotypic plasticity, Transcriptomics, Transcriptional frontloading

## Abstract

We investigated the potential for cross-generational plasticity to influence how offspring respond to hypoxia and ocean acidification (hereafter HypOA) in the coastal forage fish Atlantic silverside (*Menidia menidia*). Mature wild silversides were treated with a control [dissolved oxygen (DO):100% air saturation (a.s.)/*P*_CO_2__: 650 µatm] or HypOA (DO: 40% a.s./*P*_CO_2__: 2300 µatm) conditions for 10 days prior to spawning. Their offspring were reared under both treatments in a factorial experimental design. Parental environment had minimal effects on offspring phenotype: exposure to HypOA reduced survival and developmental rates regardless of parental treatment. However, RNAseq analysis revealed that direct offspring exposure to HypOA induced substantial transcriptional changes, with 1606 differentially expressed transcripts (DETs) in larvae from control parents. These changes affected neural development, synaptic signaling, oxygen acquisition and extracellular matrix organization. In contrast, larvae from HypOA-exposed parents exhibited a muted transcriptional response to HypOA, with only four DETs. Although we did not detect a statistically significant interaction between parental and offspring environments at the gene-wise level, a gene set test supported a consistent attenuation of expression changes in offspring from HypOA-treated parents. This pattern may be consistent with transcriptional frontloading, when stress-induced changes are retained, and may modify future responses. However, because this effect did not improve offspring performance under HypOA, they are unlikely to represent an adaptive response. Instead, they may reflect non-adaptive carryover effects of parental exposure. Our findings highlight the potential for cross-generational effects to shape transcriptional plasticity, even in the absence of benefits to offspring.

## Teaser

**Summary:** Parental exposure to hypoxia and acidification does not enhance offspring tolerance to the dual stressor, but it modestly alters larval transcriptional plasticity in response to low oxygen and elevated *P*_CO_2__.

## INTRODUCTION

Coastal hypoxia and ocean acidification (hereafter HypOA) are naturally co-occurring stressors in coastal marine ecosystems, driven by shared physical and biochemical processes that simultaneously deplete dissolved oxygen (DO), elevate carbon dioxide partial pressure (*P*_CO_2__) and reduce pH (Rabalais et al., 2009; Wallace et al., 2021). Indeed, the concurrent fluctuations of DO, *P*_CO_2__ and pH are defining characteristics of productive, shallow marine habitats (Baumann and Smith, 2017). Human-induced factors such eutrophication and habitat degradation have increased the severity, duration and spatial scale of HypOA in many coastal areas, and global warming and ocean acidification will exacerbate this trend (Cai et al., 2021; Keeling et al., 2010; Long and Mora, 2023). Nearshore marine ecosystems are an important spawning and nursery habitat for many coastal fishes and the deleterious effects of hypoxia on reproductive and developmental processes have been extensively studied (Richards, 2009). Until recently, this field largely overlooked the co-occurring nature of hypoxia and acidification in aquatic ecosystems (Gobler and Baumann, 2016). While exposure to low DO generally has stronger physiological effects than elevated *P*_CO_2__, multi-stressor experiments have revealed complex interactive effects (DePasquale et al., 2015; Hancock and Place, 2016; Yorifuji et al., 2024). Combined HypOA exposure also triggers unique transcriptional responses that cannot be predicted from individual exposures alone (Cline et al., 2020; Iguchi et al., 2024), underscoring the need for combined-stressor studies to assess organismal sensitivities to worsening HypOA in nature (Breitburg et al., 2019).

For many species, reproduction is a key sensitivity bottleneck to environmental change, making cross-generational plasticity a potentially important factor in how populations respond to emergent stressors (Byrne et al., 2020; Hackerott et al., 2021). Cross-generational plasticity describes how the environmental experiences of a parent can influence the phenotype of their offspring through various ‘non-genetic’ factors of inheritance (Adrian-Kalchhauser et al., 2020). In fish, these factors include classically described maternal effects, such as the provisioning of eggs with nutrients, hormones, proteins, mRNA and other cytoplasmic components that can influence offspring development (Green, 2008). Inheritable epigenetic factors, including DNA methylation, histone modifications and small noncoding RNAs, can be transmitted through both maternal and paternal germlines and influence offspring gene expression and responses to environmental stress (Ben Maamar et al., 2018; Kelley et al., 2021; Ord et al., 2020). Cross-generational plasticity may have evolved as a fitness-enhancing strategy in organisms that reproduce in variable environments, enabling parents to anticipate conditions offspring are likely to encounter and provide a developmental advantage (Bonduriansky, 2021). For example, treating adult zebrafish with hypoxia prior to spawning increases the acute hypoxia tolerance of offspring (Ho and Burggren, 2012; Ragsdale et al., 2022). Such a capability would be advantageous in the shallow, slow-moving waters of the natural zebrafish habitat, where diurnal hypoxia is common (Spence et al., 2008). Similarly, damselfishes experience natural *P*_CO_2__ fluctuations in coral reef habitats, and beneficial cross-generational effects to ocean acidification have been observed in multiple species (Miller et al., 2012; Monroe et al., 2021).

Transcriptional frontloading may be a useful framework for conceptualizing how parental effects alter gene expression patterns in offspring. It defines a type of acclimatory gene expression that occurs when exposure to a priming stressor elicits a persistent shift in the expression of stress response genes, enabling the organism to initiate a more rapid and efficient adaptive response upon subsequent challenges within its lifetime (Barshis et al., 2013; Hackerott et al., 2021). For example, reef-building corals (*Acropora hyacinthus*) from thermally variable environments show a frontloading of genes involved in thermal stress and exhibit a greater tolerance to bleaching compared with conspecifics from deeper, more thermally stable environments (Barshis et al., 2013). Likewise, juvenile Pacific geoduck clams (*Panopea generosa*) that were exposed to elevated *P*_CO_2__ during early development showed a frontloading of genes involved in maintaining homeostatic functions under acidification and consequently were more tolerant of repeated exposures later in life (Gurr et al., 2022). However, this framework has not yet been widely applied to detecting transcriptional effects associated with cross-generational plasticity. Transcriptional frontloading of certain stress response genes could enhance offspring tolerance to environmental stress experienced during the sensitive stages of early development.

Atlantic silverside (*Menidia menidia*) is a widespread and highly abundant forage fish that inhabits coastal ecosystems along the North American eastern seaboard (Hice et al., 2012). The early life stages of Atlantic silversides have been extensively studied for their sensitivity to low DO and elevated *P*_CO_2__, both individually and combined. Hypoxia has stronger impacts on survival, growth and respiration rates (Cross et al., 2019; DePasquale et al., 2015; Schwemmer et al., 2020); however, simultaneous exposure to elevated *P*_CO_2__ can increase offspring sensitivity to hypoxia by potentially raising the critical oxygen partial pressure and triggering the earlier onset of aquatic surface respiration (Miller et al., 2016; Schwemmer et al., 2020). Silversides are an annual, asynchronous batch-spawning species that reproduce multiple times at 2 week intervals during spring and summer in nearshore environments (Middaugh, 1981). Successive spawning events expose offspring to varying environmental conditions, as nearshore systems become more susceptible to co-occurring HypOA due to rising temperatures and increased biological productivity (Baumann et al., 2015; Murray et al., 2014). The asynchronous development of oocytes means that females complete vitellogenesis and the final stages of oocyte maturation in the days just prior to spawning (Concannon et al., 2021), allowing maternal effects and potential epigenetic influences from both sexes to adjust offspring phenotypes in response to environmental changes that occur between spawning events. These traits make silversides a useful model for studying cross-generational plasticity. Interestingly, the CO_2_ tolerance of wild silverside offspring increases seasonally, coinciding with the natural seasonal acidification of their spawning habitat (Baumann et al., 2018; Murray et al., 2014), though the mechanism underpinning this effect is unclear.

**Fig. 1.**
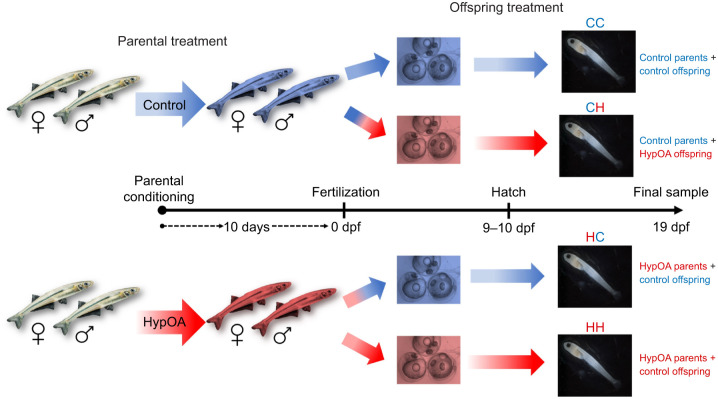
**Schematic diagram of the experimental design.** Wild mature Atlantic silversides were exposed to control or a combined hypoxia and acidification (HypOA) treatment. After fertilization, their embryos were split and reared under both treatments in a factorial design to create four offspring treatment groups: control-treated parents and control-reared offspring (CC), control-treated parents and HypOA-reared offspring (CH), HypOA-treated parents and control-reared offspring (HC) and HypOA-treated parents and HypOA-reared offspring (HH). The timeline in the center of the figure outlines the progression of the experimental phases: parental conditioning phase (10 day exposure), fertilization (0 days post-fertilization, dpf), embryo phase (0 to 9 or 10 dpf), and larval rearing phase (9 or 10 to 19 dpf). Larvae were counted and subsampled at hatch and at 19 dpf to measure embryo and larval survival as well as morphometric traits. RNA was extracted from an additional set of larvae at 19 dpf for transcriptomic analysis.

In this study, we explored cross-generational plasticity in Atlantic silversides under a combined exposure to low DO and elevated *P*_CO_2__, mimicking the naturally occurring HypOA conditions in their spawning environment (Baumann and Smith, 2017; Baumann et al., 2015). To test our prediction that parental environment between spawning events can influence offspring tolerance to environmental stress, we exposed reproductively mature wild Atlantic silversides for 10 days to either control conditions or a HypOA treatment. We reared their offspring in a factorial experimental design that produced four offspring treatment groups (Fig. 1). We monitored offspring survival and growth over the first 19 days of embryo and larval development, the period during which notochord flexion is typically completed, to test the prediction that parental exposure to HypOA would reduce the negative effects of direct HypOA exposure. Additionally, we used bulk RNA sequencing to examine gene expression in larvae, evaluating the prediction that cross-generational effects would involve the frontloading of genes associated with functional pathways promoting HypOA tolerance.

List of abbreviations

a.s.: air saturation
*A*_T_: total alkalinity
BP: biological process (gene ontology)
CC: control-treated parents and control-reared offspring
CER: constitutive expression ratio
CH: control-treated parents and HypOA-reared offspring
CPM: counts per million
DET: differentially expressed transcript
DIC: dissolved inorganic carbon
DO: dissolved oxygen
dpf: days post-fertilization
dph: days post-hatch
FC: fold-change
FDR: false discovery rate
FRC: fold-change ratio
GABA: gamma-aminobutyric acid
GO: gene ontology
HC: HypOA-treated parents and control-reared offspring
HH: HypOA-treated parents and HypOA-reared offspring
Hif: hypoxia induced factor
HypOA: concurrent hypoxia and acidification
IVF: *in vitro* fertilization
MF: molecular function (gene ontology)
*P*_CO_2__: partial pressure of CO_2_
pH_T_: pH on the total scale

## MATERIALS AND METHODS

### Experimental treatments

We implemented two experimental treatment levels: a control treatment with DO set to 100% air saturation (a.s.; or 7.3 mg l^−1^) combined with 670 µatm *P*_CO_2__ (7.83 pH total scale, pH_T_), and a HypOA treatment with a DO at 40% a.s. (or 3.0 mg l^−1^) combined with 2300 µatm *P*_CO_2__ (7.33 pH_T_). The control *P*_CO_2__ is higher than atmospheric and typical open ocean *P*_CO_2__ (∼430 µatm) but reflects the typical conditions observed in the productive nearshore systems where Atlantic silversides reproduce (Baumann et al., 2015). Conversely, the HypOA treatment is based on long-term monitoring of co-varying pH and DO in a coastal salt marsh and reflects conditions during periods of intense net heterotrophy (Baumann et al., 2015; Cross et al., 2019). Past studies have shown that this specific combination of low DO and elevated *P*_CO_2__ moderately reduces the survival, growth and oxygen consumption rates in Atlantic silverside embryos and larvae but does not cause the near-total mortality seen in more extreme HypOA conditions, which would complicate interpreting parental effects (Cross et al., 2019; Schwemmer et al., 2020). A seawater temperature of 21°C was used for all experimental protocols, as this matches the seasonal temperature at the collection site of wild adult Atlantic silverside and is close to the optimal temperature for the growth and survival of their offspring (Murray and Baumann, 2018). Note that offspring were exposed to static experimental conditions, whereas adults experienced fluctuating treatment conditions, as described below.

### Description of the experimental system

The experiment was conducted at the Environmental Systems Laboratory (ESL) facility at the Woods Hole Oceanographic Institution (WHOI) in an automated experimental seawater system composed of eight 900 l circular tanks (four tanks each for control and HypOA treatment) and one 600 l mixing tank. A schematic diagram of the experimental setup is shown in Fig. S1. Each 900 l tank was outfitted with multiple 20 l rearing containers (white buckets fitted with six 3 cm flowthrough holes covered in 300 µm screening) to accommodate embryos and larvae. The four control tanks received temperature-controlled seawater (21°C) sourced from Vineyard Sound (sand-filtered, UV-sterilized and maintained at the control treatment DO and *P*_CO_2__). HypOA treatment conditions were maintained within the mixing tank using custom software developed in Arduino IDE and operated by a Teensy 4.1 Development Board. Briefly, the mixing tank received a continuous flow of ambient seawater, with DO, pH and temperature measured every 10 s by Pyroscience Ultra Compact (PICO) DO and pH meters, fitted with fiberoptic DO or pH sensors and PT100 temperature sensors. Calibration of the probes was performed weekly according to the manufacturer's guidelines using oxygen and pH_T_ calibration solutions provided by Pyroscience. HypOA levels were regulated through a simple feedback mechanism: when the current DO or pH exceeded the treatment set point, the software opened a solenoid valve to introduce compressed gas (N_2_ or CO_2_) into ultrafine gas-diffusers positioned at the bottom of the mixing tank until the setpoint was reached. The software was optimized to implement brief and precisely controlled gas additions, ensuring stable DO and pH conditions in the mixing tank. Adjusted HypOA seawater was continuously pumped to the main tanks and individual rearing containers.

DO levels in individual rearing units were cross-verified using a handheld Pyroscience Firesting-GO2 meter fitted with an optical DO sensor. A two-point calibration (100% and 0% DO) was performed daily using air-saturated seawater and a concentrated NaSO_3_ seawater solution. pH conditions were verified via a Hach HQ11D pH meter fitted with a Hach PHC28101 IntelliCal pH probe. The probe was calibrated daily using pH_T_ buffers from PyroScience. To validate target *P*_CO_2__ conditions, seawater was sampled every 1–2 days from one of the replicate rearing containers per offspring treatment group, resulting in 12 seawater samples per offspring treatment group during the 19 day experiment. Seawater samples were sterilized with mercuric chloride and stored in sealed bottles for later analysis of carbon chemistry parameters. Temperature and salinity (via refractometer) were recorded at the time of sampling. Samples were measured in triplicate for dissolved inorganic carbon (DIC) using an Apollo AS-D1 analyzer connected to a Picarro G-2121i cavity ringdown system. Total alkalinity (*A*_T_) was measured using an open-system Gran titration on 5 ml samples in triplicate, using a Metrohm 805 Dosimat and a robotic Titrosampler. Both systems were calibrated against seawater-certified reference materials (Dickson, 2024). The remaining *in situ* carbon chemistry parameters of pH_T_, *P*_CO_2__, bicarbonate and carbonate ions were calculated using the R package *seacarb* (https://cran.r-project.org/package=seacarb) based on direct measurements of DIC, *A*_T_ and the salinity and temperature at the time of water sampling. Equilibrium constants for the dissociation of carbonic acid in seawater (*K*_1_ and *K*­_2_) followed Mehrbach et al. (1973), refitted by Dickson and Millero (1987). The constant for KHSO_4_ followed Dickson (1990). Measured and derived carbon chemistry parameters are listed in Table 1.

**Table 1.** Summary of treatment conditions

Group	Temperature (°C)	DO (% a.s.)	pH_T_ (spot check)	DIC (μmol kg^−1^)	*A*_T_ (μmol kg^−1^)	*P*_CO_2__ (μatm)	pH_T_ (calculated)
CC	21.0±0.4	100.2±2.1	7.80±0.06	1962±16	2104±13	671±53	7.82±0.03
CH	21.1±0.4	40.6±9.9	7.33±0.05	2115±14	2101±11	2253±203	7.34±0.04
HC	21.0±0.4	100.3±1.9	7.79±0.07	1964±20	2106±12	671±77	7.83±0.05
HH	21.1±0.4	40.1±7.3	7.32±0.04	2117±14	2101±12	2304±233	7.33±0.05

Values are presented as offspring treatment means (±s.d.). Temperature, dissolved oxygen (DO; % air saturation, a.s.) and pH on the total scale [pH_T_ (spot check)] were obtained from daily spot check measurements using handheld dip probes (*N*=20). Dissolved inorganic carbon (DIC) and total alkalinity (*A*_T_) were measured directly from preserved seawater samples (*N*=12). *P*_CO_2__ and pH_T_ (calculated) were calculated from measured DIC, *A*_T_, temperature and salinity.

### Wild fish collection and care

Animal welfare protocols and experimental procedures followed guidelines set forth by WHOI's Institutional Animal Care and Use Committee (WHOI protocol #28188). Wild adult Atlantic silversides, *Menidia menidia* (Linnaeus 1766), were collected on 31 May 2022 by beach seine (30×2 m) from a single location in Mumford Cove, CT, USA, a small seagrass-dominated embayment connected to eastern Long Island Sound (41.32°N, 72.02°W). Fish were transported in an aerated cooler to the ESL where they were separated by sex and then into two groups for treatment with either control or HypOA conditions (34 males and 37 females per treatment group). Adults were acclimated to laboratory conditions for 5 days in large circular tanks (one tank per sex and treatment group). Tanks received a continuous flow (5 l min^−1^) of ambient seawater. Fish were hand fed to satiation twice daily with frozen adult brine shrimp and blood worms (San Francisco Bay Brand).

### Parental acclimation and fertilization protocols

Following the initial 5 day acclimation period, adult fish were exposed to their respective experimental treatment conditions for 10 days. Parental treatment with HypOA involved exposure to a fluctuating DO and *P*_CO_2__ regime [control treatment: 07:00–19:00 h; HypOA treatment: 19:00–07:00 h], mimicking the diurnal fluctuations and nocturnal hypoxia and acidification that characterize nearshore marine environments (Baumann et al., 2015). Flowrates to all adult tanks were maintained at 5 l min^−1^ and continuous DO measurements of adult HypOA tanks and DO and pH conditions in the mixing tank are displayed in Fig. S2. We implemented a fluctuating HypOA protocol over a static treatment out of concerns that chronic exposure to HypOA conditions might strongly affect reproductive success. Adults were hand-fed frozen brine shrimp and blood worms to satiation twice daily. Following acclimation, separate *in vitro* fertilizations (IVF) were conducted for each parental group, following established protocols (Murray and Baumann, 2018; Murray et al., 2017). These methods are optimized to produce maximum genetic diversity within experimental replicates (Malvezzi et al., 2015). For each parental group, 17–20 males and 7 females contributed gametes during fertilization. Their lengths and mass are reported in Table S1. Embryos were screened within 1 h of IVF and viable embryos were selected for rearing.

### Offspring rearing and sampling

Embryos were reared in 500 ml plastic cups with 300 µm screened bottoms that were floated inside the 20 l rearing containers. For calculating survival traits, 50 embryos were allotted to replicate rearing cups (*N*=4 replicate cups per offspring treatment group). For measuring growth and transcriptomic analysis, an additional rearing cup was allotted 200 embryos to serve as a source for subsamples (*N*=4 replicate cups per offspring treatment group). See Fig. S1 for an overview of the experimental setup. Seawater lines were positioned directly inside all embryo cups to provide a direct seawater flow over embryos (30 ml min^−1^). Initially, all rearing containers received a flowthrough of control seawater and the introduction of HypOA-adjusted seawater into designated replicates began 4 h post-fertilization. Static experimental conditions were maintained for the duration of the experiment. Spot-check measurement of all rearing containers for DO, pH and temperature was carried out at least once daily using handheld GO2 and Hach pH meters. A summary of the spot-check measurements is provided in Table 1.

Starting 6 days post-fertilization (dpf), replicates were checked in the morning for newly hatched larvae. Hatchlings from survival replicates were counted and moved to a new 20 l container to monitor post-hatch survival (flowthrough increased to 120 ml min^−1^). Subsamples for morphometric analysis at hatch (*N*=10) were collected on the first morning when a growth replicate had at least 20 new hatchlings. The larvae were euthanized by an overdose of MS-222 and were fixed in 4% neutrally buffered formalin for 24 h and transferred to 75% ethanol for storage. The remaining hatchlings from growth replicates were moved to a new 20 l container for continued rearing. Larvae were fed daily with newly hatched brine shrimp nauplii (San Francisco Bay strain) at an initial concentration of 2–3 nauplii ml^−1^. Replicate vessels were siphoned daily for uneaten food and waste. The continuous seawater flow through ensured that ammonia levels remained near 0 ppm, verified daily using an API Ammonia Test Kit. At 19 dpf [9 or 10 days post-hatch (dph) based on the treatment and hatch timing] the survival replicates were recounted to determine the final survival rate. Larvae were subsampled for morphometric measurements (*N*=12) as described above. Additionally, a subset of larvae (19 dpf) was sampled individually (*N*=6 per treatment, two larvae from three of the replicate rearing containers) and immediately snap-frozen in liquid nitrogen and stored at −80°C for subsequent RNAseq analysis. We chose to sample all larvae at the same absolute age for RNAseq, despite the likelihood that HypOA conditions would result in smaller, less developed larvae. This approach ensured consistency with other measured traits and minimized potential confounding factors associated with varying absolute ages.

Formalin-preserved larvae were laterally photographed using a digital camera attached to a dissecting microscope. Morphometric measurements were made using ImageJ2 (v.1.53; Rueden et al., 2017). Newly hatched larvae were measured for four traits: standard length (tip of the snout to the end of the notochord; nearest 0.01 mm), eye diameter (anterior to posterior diameter of the left eye, 0.01 mm), body depth (myomere height immediately posterior to the anus, 0.01 mm) and yolk sac profile area (0.01 mm^2^). The larvae sampled at 19 dpf were measured for standard length, body depth and eye diameter.

### RNA extraction and sequencing

Total RNA was extracted from individual larvae (*N*=6 per offspring treatment group; 4 treatments) using the Qiagen RNeasy Mini extraction kit following the manufacturer’s guidelines. RNA purity was confirmed using a Nanodrop 2000 Spectrophotometer and total RNA concentration was quantified using a Qubit fluorometer. The RNA samples were shipped on dry ice to Novogene Inc. (Sacramento, CA, USA), where quality was assessed using Agilent Bioanalyzer (RIN>9.3 for all samples). An NEBNext Ultra™ II Directional RNA Library Prep Kit was used for library generation and sequencing was carried out on a NovaSeq 6000 platform (150 bp paired-end reads) at a sequencing depth of 20 million reads per sample.

### Bioinformatics

Initial data processing involved trimming reads to remove adapter sequences, reads with unknown bases and low-quality reads using the default settings for paired-end data in Fastp v.0.23.4 (Chen et al., 2018). Sequencing produced a mean±s.d. of 17.74±1.39 million clean reads per sample. The STAR v.2.6.1d tool (Dobin et al., 2013) was used to align reads to a high-quality *M. menidia* reference genome (Jacobs et al., 2024). Gene count quantification for each sample was performed using HTseq-count v.0.11.1 (Anders et al., 2015). On average, 73.0±1.2% of clean reads were mapped to the *M. menidia* genome across samples, resulting in the annotation of 17,263 unique coding sequences (transcripts). We focused our analysis on 15,517 transcripts with annotations for NCBI gene symbols and Gene Ontology (GO) terms (The Gene Ontology Consortium, 2018).

### Statistical analysis of survival and growth traits

Statistical procedures were completed using R (v.4.0.2) in RStudio (v.1.3). Model performance and adherence to assumptions were confirmed using the *Performance* package (https://CRAN.R-project.org/package=performance). Statistical significance was set at α=0.05. Results are presented as treatment means±s.d. unless specified otherwise. Time (days) to peak hatch was calculated for each replicate (i.e. the day with the highest hatch count). Replicate survival was quantified for two intervals: embryo survival (ratio of total hatch count over the initial 50 embryos) and larval survival (ratio of larvae surviving at 19 dpf over initial hatch count). Ratio data were logit transformed prior to testing (Warton and Hui, 2011). The individual and interactive effects of offspring×parental treatment conditions on peak hatch and survival traits were examined using two-way analysis of variance (ANOVA).

The individual and interactive effects of offspring×parental treatments on standard length at hatch, yolk sac area at hatch and standard length at trial termination were analyzed using mixed-effects ANOVA (Satterthwaite's degrees of freedom method) using the *lme4* and *lmerTest* R packages (Bates et al., 2015; Kuznetsova et al., 2017). Replicate ID was set as a random factor to account for the shared environment of larvae sampled from the same rearing container. We calculated size-independent indices for body depth and eye width. Standardized residuals were derived from a common linear regression fit with standard length using all samples pooled across treatments for each age group (Fig. S3). We used mixed-effect ANOVA, as described above, to test treatment effects on normalized body depth and normalized eye width for larvae of each age. A larval growth rate (mm day^−1^) was computed for each subsample replicate as the average final standard length minus the average hatch standard length divided by the growth interval in days. Treatment effects on larval growth rates were analyzed by two-way ANOVA. *Post hoc* multiple-comparison tests were conducted using Tukey's HSD via the R package *emmeans* (https://CRAN.R-project.org/package=emmeans).

### Statistical analysis of RNAseq data

Aligned gene counts from all samples were assembled into a gene-level DGEList, filtered, and normalized using the R package *edgeR* v.3.40.2 (Robinson et al., 2010). Lowly expressed genes were filtered out using the function filterByExpr (default parameters). Raw counts were normalized (calcNormFactors function, TMM method) and PERMANOVA (1×10^6^ permutations) was used to test significant effects of parental and offspring HypOA exposure on global gene expression. Sample ordination was visualized using PCoA (Manhattan distances) in the *vegan* package (https://github.com/vegandevs/vegan). Differentially expressed transcripts (DETs) between each pairwise treatment comparison were identified using gene-wise negative binomial generalized linear models, implemented with the glmQLFit and glmQLFTest functions. We used this model to identify genes significantly affected by the interaction between parental and offspring environments. Significance of DETs was determined at a false discovery rate (FDR) of <0.05. Furthermore, we used the CAMERA function in edgeR, a competitive gene set test (Wu and Smyth, 2012), to test whether genes affected by offspring HypOA exposure in one parental group exhibited a statistically similar response in the other. To explore potential evidence of transcriptional frontloading in larvae from HypOA-treated parents, we conducted an exploratory analysis using methods adapted from Barshis et al. (2013). Additional details and results of this analysis are provided in the Appendix.

To determine the functional patterns associated with changes in gene expression, we assessed DETs from different sets of pairwise comparisons for significantly enriched (adjusted *P*<0.10) GO biological process (BP) and GO molecular function (MF) terms using the enricher function in *ClusterProfiler* v.4.6.2 (Wu et al., 2021). The background gene list included all expressed transcripts in the filtered DGEList. Redundancy reduction amongst enriched terms was carried out using the R package *rrvgo* (Sayols, 2023). A similarity matrix was generated based on the frequency of shared genes between GO terms using the calculateSimMatrix function (Wang method) and similar terms were clustered into groups using the reduceSimMatrix function (similarity threshold of 0.7). Parent terms for each cluster were chosen based on the term with the lowest *P*-value. Heatmaps were plotted using the R package *complexHeatmap* (Gu et al., 2016).

## RESULTS

### Survival and hatching

**Fig. 2.**
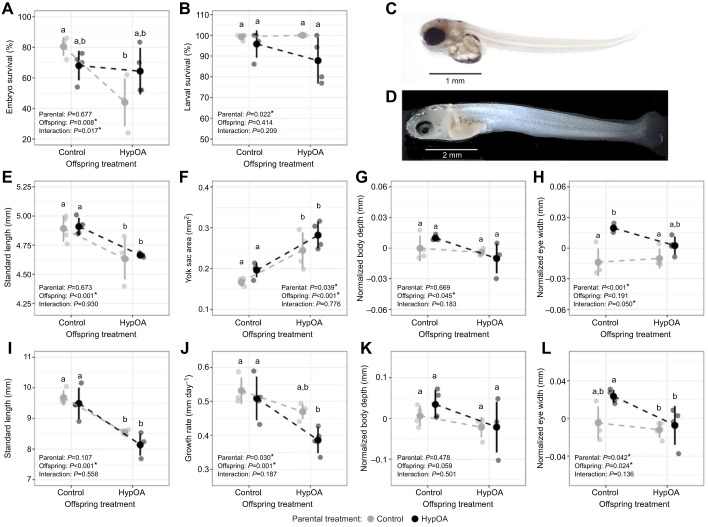
**Treatment effects on offspring survival and morphometric traits.** (A) Embryo survival (fertilization to hatch, *N*=4) and (B) post-hatch larval survival (hatch to 19 dpf, *N*=4). Images of (C) newly hatched larvae (9 dpf) and (D) larvae at 19 dpf or 10 days post-hatch. (E) Standard length at hatch (*N*=10); (F) yolk sac profile area at hatch (*N*=10); (G) normalized body depth at hatch (*N*=10); (H) normalized eye width at hatch (*N*=10); (I) final standard length (*N*=12); (J) post-hatch growth rate (*N*=4); (K) final normalized body depth (*N*=12); and  (L) final normalized eye width (*N*=12). Large circles show treatment mean values and vertical lines indicate ±s.d. calculated from treatment mean values. Small circles show replicate mean values. Dashed lines show reaction norms by parental treatment. Significance levels (*P-*values) are presented from two-way ANOVA (survival and growth rate) or two-way mixed effect ANOVA (morphometric traits) testing the effects of parental and offspring treatments and their interaction. Differing letters above or below each group indicate significant pairwise differences in survival (Tukey's HSD, *P<*0.05).

Embryo survival was significantly affected by a parental×offspring interactive effect (ANOVA, *P=*0.017; Fig. 2A). HypOA exposure significantly reduced the survival of embryos from control parents (Tukey's HSD, *P*=0.006; Fig. 2A). However, there was no difference in the survival rates of embryos reared under HypOA and control conditions from HypOA-treated adults (Tukey's HSD, *P=*0.9901; Fig. 2A). Overall, embryo survival from HypOA-treated adults fell between the survival rates of the CC and CH groups. Consequently, there was no significant difference in survival between parental groups within each offspring treatment (Fig. 2A). Time to peak hatch was 9 dpf in embryos reared under the control treatment and this was delayed by 1 day (10 dpf) in embryos exposed to HypOA (ANOVA, *P=*0.001). Parental treatment had no effect on time to hatch. Larval survival was generally high and not significantly affected by offspring treatment but was significantly lower in offspring from HypOA-exposed parents when averaged across offspring treatments (ANOVA, *P*=0.022; Fig. 2B).

### Growth

Embryos reared under HypOA were 5.2% shorter at hatch (mixed-effect ANOVA, *P*<0.001; Fig. 2E) and had yolk sacs that were 42% larger than those of control-reared embryos (mixed-effect ANOVA, *P*<0.001; Fig. 2F). Parental treatment did not influence standard length of hatchlings (Fig. 2E). Yolk sacs were 14% larger in hatchlings from HypOA-treated parents when averaged across offspring treatments (mixed-effect ANOVA, *P*=0.039), but pairwise comparisons did not find significant differences within offspring treatment (Fig. 2F). Normalized body depth at hatch was significantly smaller in offspring reared under HypOA (mixed-effect ANOVA, *P*=0.045), but no significant differences were observed between individual treatment groups (Fig. 2G). On average, normalized eye width was significantly larger in hatchlings from HypOA-treated parents (mixed-effect ANOVA, *P*<0.001; Fig. 2H). This effect was primarily driven by the relatively large eyes exhibited by the HC group (Fig. 2H), indicating a potential parental×offspring treatment interaction, though the effect was not significant (mixed-effect ANOVA, *P*=0.051).

At trial termination (19 dpf), larvae reared under HypOA were 12.9% shorter in length compared with larvae reared under control conditions (mixed-effect ANOVA, *P<*0.001; Fig. 2I). This resulted in a 17.7% reduction in post-hatch growth rate in comparison to larvae reared in control conditions (mixed-effect ANOVA, *P=*0.001; Fig. 2J). Additionally, the average growth rate of larvae from HypOA-treated parents was 10.8% lower relative to that of larval groups from control parents (mixed-effect ANOVA, *P=*0.030; Fig. 2J). The interactive effect on growth rate was not significant, but the additive effect of parental and offspring HypOA exposure meant that HH larvae displayed the slowest overall larval growth, though not significantly different from that of CH larvae (Tukey's HSD, *P=*0.076; Fig. 2J). Normalized body depth was not statistically different between treatment groups (Fig. 2K). However, normalized eye width was significantly affected by both parental treatment (mixed-effect ANOVA, *P=*0.042) and offspring treatment (mixed-effect ANOVA, *P=*0.024; Fig. 2L). This effect was primarily driven by the HC group, which exhibited larger eyes on average compared with all other groups (Fig. 2L).

### Treatment effects on global gene expression

**Fig. 3.**
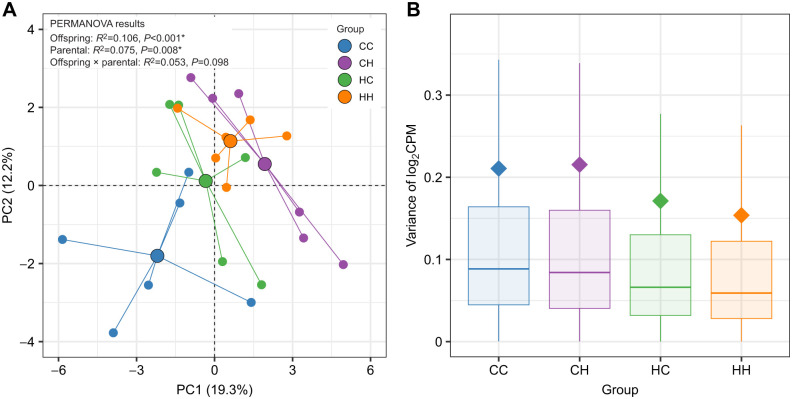
**Treatment effects on global gene expression.** (A) Results from principal coordinate (PC) analysis. Small colored circles represent the coordinates of individual samples (*N*=6), and they are connected to large colored circles, which represent the average coordinate position for each treatment group. The distances between points reflect the similarity in gene expression profiles among individual larvae and treatments. The results of a two-way PERMANOVA are presented in the top left of the panel, demonstrating the influence of both parental and offspring treatment conditions on the gene expression patterns observed. (B) Gene-wise variance of log_2_ counts per million (CPM) across treatment groups. Colored boxes indicate the interquartile range, while the vertical lines extend to 1.5 times the interquartile range. The horizontal bar represents the median value for each treatment, and the colored diamond denotes the mean values. For clarity, individual variance values that fall outside the range are not shown in the plot.

Analysis of RNAseq data with two-way PERMANOVA showed that global gene expression in silverside larvae was affected by both offspring (*P<*0.001, *R*^2^=0.106) and parental treatment conditions (*P=*0.008, *R*^2^=0.075; Fig. 3A). There was no interactive effect (Fig. 3A). Ordination using PCoA revealed distinct separation among the four treatment groups (Fig. 3A). Larvae reared in HypOA (CH and HH) were clustered away from the CC group, whereas larvae from the HC group replicates were positioned between the other clusters (Fig. 3A). In general, offspring from HypOA-treated parents clustered more closely than those from control parents (Fig. 3A). Intra-treatment transcript-wise variation in expression was greater within the CC and CH groups as compared with HC and HH larvae (Fig. 3B).

### Pairwise treatment tests of differential expression

**Fig. 4.**
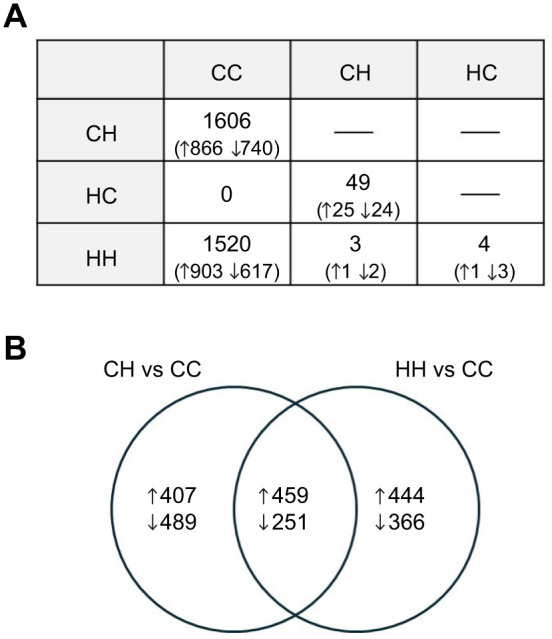
**Summary of differentially expressed transcripts (DETs) from pairwise comparisons between treatment groups.** (A) Table showing the number of DETs for all six pairwise comparisons between the four offspring treatment groups (*N*=6 larval samples per group): CC, CH, HC and HH. The column headers (CC, CH, HC) and row headers (CH, HC, HH) represent the different treatment groups. The value in each cell represents the number of DETs identified in the pairwise comparison between the respective row and column treatment groups. Numbers in parentheses indicate the count of upregulated and downregulated transcripts (indicated by up and down arrows) in relation to the treatment group listed in the row header. (B) Venn diagram showing the number of shared and unique DETs between the CH versus CC and HH versus CC pairwise comparisons. Upregulated and downregulated genes (indicated by up and down arrows) are shown separately**.**

Pairwise comparisons between the overall control group and the two groups that were directly exposed to HypOA revealed similar numbers of DETs. The CH versus CC comparison found 1606 DETs, while the HH versus CC comparison identified 1520 DETs (Fig. 4A). Notably, over half of the DETs in the HH versus CC comparison differed from those found in the CH versus CC comparison (Fig. 4B). In contrast, fewer DETs were detected when comparing the groups reared in HypOA with the larval group obtained from HypOA-treated parents and reared under control conditions, with only 4 DETs detected in the HH versus HC comparison and 49 DETs in the CH versus HC comparison (Fig. 4A). The two groups that were directly exposed to HypOA (HH versus CH) showed 3 DETs, and no DETs were found between the groups that were directly reared under the control treatment (HC versus CC; Fig. 4A). Despite differences in DETs in response to offspring HypOA exposure between parental treatments, no transcripts were identified as significantly affected by a parental×offspring interaction. Volcano plots for each pairwise comparison are shown in Fig. S4. Dataset 1 includes all differential expression results.

### Functional enrichment of differentially expressed transcripts

**Fig. 5.**
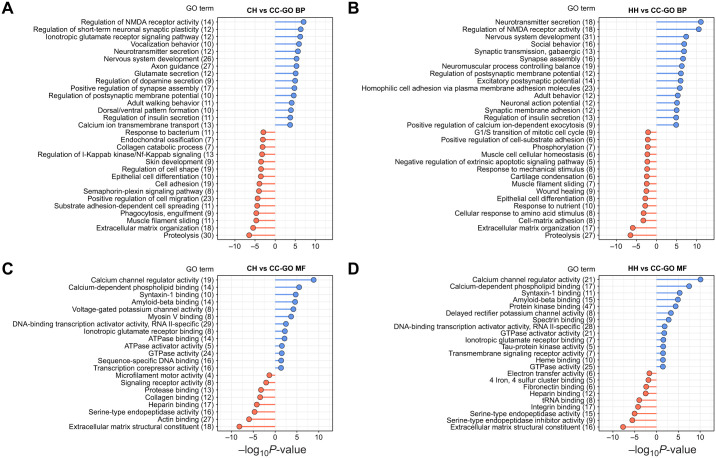
**GO ‘biological process’ (BP) and ‘molecular function’ (MF) terms enriched in DETs expressed by CH and HH larvae.** The length of the segment corresponds to the significance of the term (−log_10_*P*-value) for upregulated (blue) and downregulated (red) DETs. Downregulated terms are shown with negative significance values for clarity. Values in parentheses next to term names indicate the number of transcripts contributing to the enrichment.

We performed GO enrichment analysis on transcripts from the two pairwise comparisons with the highest number of DETs (CH versus CC and HH versus CC) to better understand how parental treatment influenced the transcriptional response to HypOA. Upregulated and downregulated DETs from the CH versus CC comparison were enriched in 88 GO BP and 60 GO MF terms. DETs from the HH versus CC comparison were enriched in 73 GO BP and 25 GO MF terms (see Dataset 1 for detailed results). While the majority of significantly enriched GO terms were unique to either CH or HH, the most highly enriched terms were conserved between the two groups (Fig. 5). For example, upregulated DETs from both groups were enriched in GO BP terms for neurotransmitter secretion, nervous system development, regulation of NMDA receptor activity, ionotropic glutamate receptor signaling pathway and regulation of postsynaptic membrane potential (Fig. 5A,B). Top GO MF terms in both groups included calcium channel regulator activity, calcium-dependent phospholipid binding, syntaxin-1 binding and potassium channel activity (Fig. 5C,D). One difference was the enrichment of terms related to GABAergic and glutamatergic synaptic transmission in HH larvae, as several transcripts that regulate the expression of NMDA and gamma-aminobutyric acid (GABA) receptor subunits were significantly upregulated by HH larvae only (Fig. 5A,C). A similar pattern was found for downregulated DETs, in that the specific GO terms varied between groups, but the most highly enriched terms were shared between CH and HH larvae (Fig. 5). For example, the most enriched GO BP terms for both groups included proteolysis, extracellular matrix organization, positive regulation of cell migration and wound healing (Fig. 5A,B). Shared GO MF terms included extracellular matrix constituent, serine-type endopeptidase activity and heparin binding (Fig. 5C,D). Interestingly, the downregulated DETs in the CH group were enriched in unique immune response terms not found in the HH group, such as phagocytosis, response to bacteria and viral entry into host (Dataset 1).

### Effect of parental environment on larval transcriptional plasticity

**Fig. 6.**
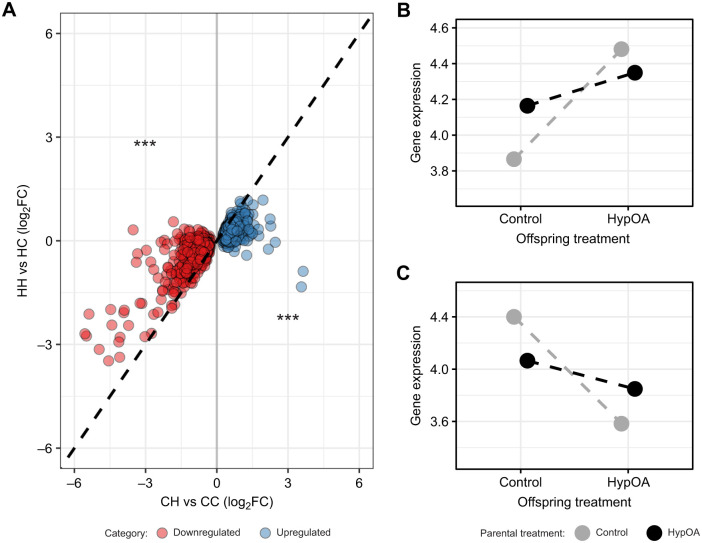
**Parental effects on transcriptional plasticity.** (A) Comparison of log­_2_ fold-change (log_2_FC) values between the CH versus CC and HH versus HC pairwise comparisons for the 1603 DETs identified in the CH versus CC comparison. DETs with equal log_2_FC values in the two comparisons should align with the equality line (dashed black line). Upregulated DETs (blue circles) with higher log_2_FC in the CH versus CC comparison fall below this line, while downregulated DETs (red circles) with greater log_2_FC fall above it. Asterisks indicate a significant effect detected by CAMERA test for upregulated (*P*=3.485×10^−66^) and downregulated transcripts (*P=*7.704×10^−55^). Transcriptional reaction norms for upregulated (B) and downregulated (C) DETs. Gray and black circles indicate mean expression (log_2_CPM) for each treatment group, while dashed lines represent transcriptional reaction norms based on parental treatment.

To better understand the attenuated transcriptional response to HypOA in larvae from HypOA-treated parents (i.e. the reduced number of DETs), we focused on the 1606 DETs identified in the CH versus CC comparison, which represents the core set of larval transcripts responsive to direct HypOA exposure. To test whether this response was consistent across parental treatments, we used the CAMERA gene set test to evaluate whether genes differentially expressed in the CH versus CC contrast showed similar expression changes in the HC versus HH comparison. Genes upregulated in CH versus CC (*N*=866) showed significantly reduced upregulation or reversal to downregulation in the HC versus HH comparison (*P*=3.48×10^−66^). Likewise, genes downregulated in CH versus CC (*N*=740) exhibited significantly attenuated downregulation or partial reversal in HC versus HH (*P*=7.70×10^−55^). These results indicate that parental HypOA exposure substantially attenuated offspring transcriptional plasticity in response to HypOA (Fig. 6A). When plotted as reaction norms, upregulated DETs exhibited a shallower slope in larvae from HypOA-treated parents (Fig. 6B). This was driven by higher expression in HC versus CC larvae and lower expression in HH versus CH larvae. A similar but inverse pattern was observed for downregulated DETs (Fig. 6C). HC larvae showed lower mean expression than CC larvae, while HH larvae exhibited higher mean expression than CH larvae. These transcripts were subsequently examined for patterns of transcriptional frontloading, with results presented in the Appendix.

## DISCUSSION

### Offspring from HypOA-treated parents were not more HypOA tolerant and showed reduced transcriptional plasticity

The increasing severity of HypOA threatens fish populations that depend on productive nearshore environments for reproduction (Gobler and Baumann, 2016). In this study, we explored whether cross-generational plasticity in Atlantic silversides, a coastal forage fish that spawns in shallow nearshore habitats, can influence offspring sensitivity to this combined stressor. We exposed wild-caught, mature Atlantic silversides to either a control or HypOA treatment for 10 days between their bi-weekly spawning events and then reared their offspring under both treatment conditions in a factorial experimental design. Offspring exposed to HypOA showed reduced embryo survival and slower embryonic and larval developmental rates. Importantly, parental exposure to HypOA did not increase offspring tolerance, as survival and growth patterns under HypOA were largely similar between parental treatments. RNAseq analysis revealed that the parental environment had a subtle but consistent effect on larval transcriptional patterns. Larvae from control parents showed pronounced gene expression changes in response to HypOA, with 1606 DETs identified between CH and CC groups. In contrast, larvae from HypOA-treated parents exhibited minimal transcriptional differences between HH and HC groups, with just 4 DETs. This attenuation was not due to a general insensitivity to HypOA, as the HH group still had 1520 DETs compared with the overall control (CC) group. These results suggest that parental HypOA exposure dampened the differential expression typically observed under direct HypOA exposure, leading to a reduced transcriptional response between HC and HH larvae.

**Fig. A1.**
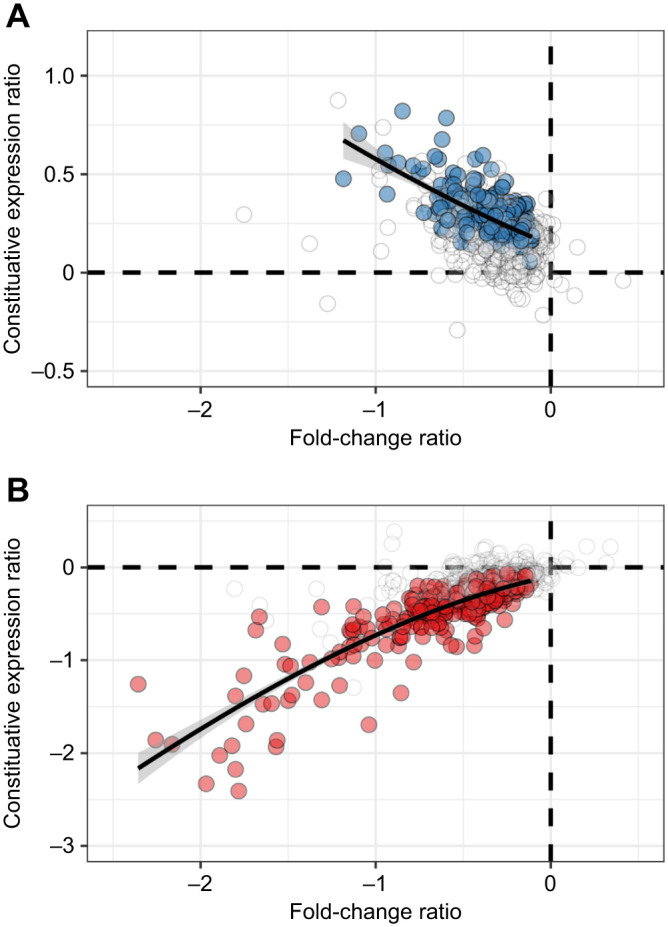
**Scatterplots comparing the fold-change ratio and constitutive expression ratio for 1603 upregulated and downregulated DETs.** Colored circles (blue for upregulated, A; red for downregulated, B) indicate DETs that met the frontloading criteria in larvae from HypOA-treated parents, while open circles represent DETs that did not meet all three criteria. A LOESS curve (solid black line) with a 95% confidence interval (shaded area) illustrates the relationship between fold-change ratio and constitutive expression ratio for frontloaded DETs, showing a larger absolute constitutive expression ratio (positive for upregulated).

**Fig. A2.**
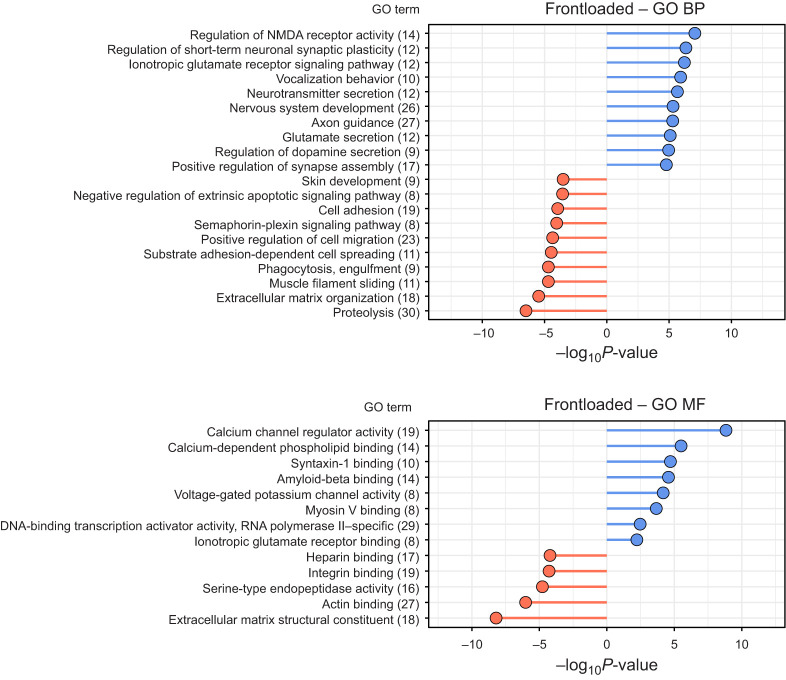
**GO biological process (BP) and molecular function (MF) terms that were enriched in frontloaded DETs.** The length of the segment corresponds to the significance of the term (−log_10_*P*-value) for upregulated (blue) and downregulated (red) genes. Downregulated terms are shown with negative significance values for clarity. Values in parentheses next to term names indicate the number of genes contributing to the enrichment of that term.

Importantly, this parental effect on gene expression was not statistically significant. Differential expression analysis did not detect any transcripts significantly affected by a parental×offspring interaction, nor were there DETs between larvae reared under identical conditions from different parental treatments. The lack of phenotypic differences between these groups further supports the conclusion that parental HypOA exposure had limited functional impact on offspring under our experimental conditions. Still, the consistent attenuation of expression across many genes involved in the core HypOA response suggests a possible signal of transcriptional frontloading, where prior stress exposure primes gene expression for a muted or more efficient response upon re-exposure (Barshis et al., 2013; Collins et al., 2021; Gurr et al., 2022). In an exploratory analysis, we identified a subset of genes showing quantitative evidence of frontloading (Fig. A1), many of which are linked to key GO BP and MF terms related to HypOA response (Fig. A2). This frontloading signal may reflect cross-generational plasticity via maternal or epigenetic mechanisms (Adrian-Kalchhauser et al., 2020; Green, 2008). However, these effects did not translate into improved offspring tolerance to HypOA within the experiment's duration. Thus, this patten may largely reflect a by-product of adult exposure that altered gene regulatory patterns in offspring, rather than an adaptive cross-generational response to HypOA.

### The transcriptional response to HypOA

**Fig. 7.**
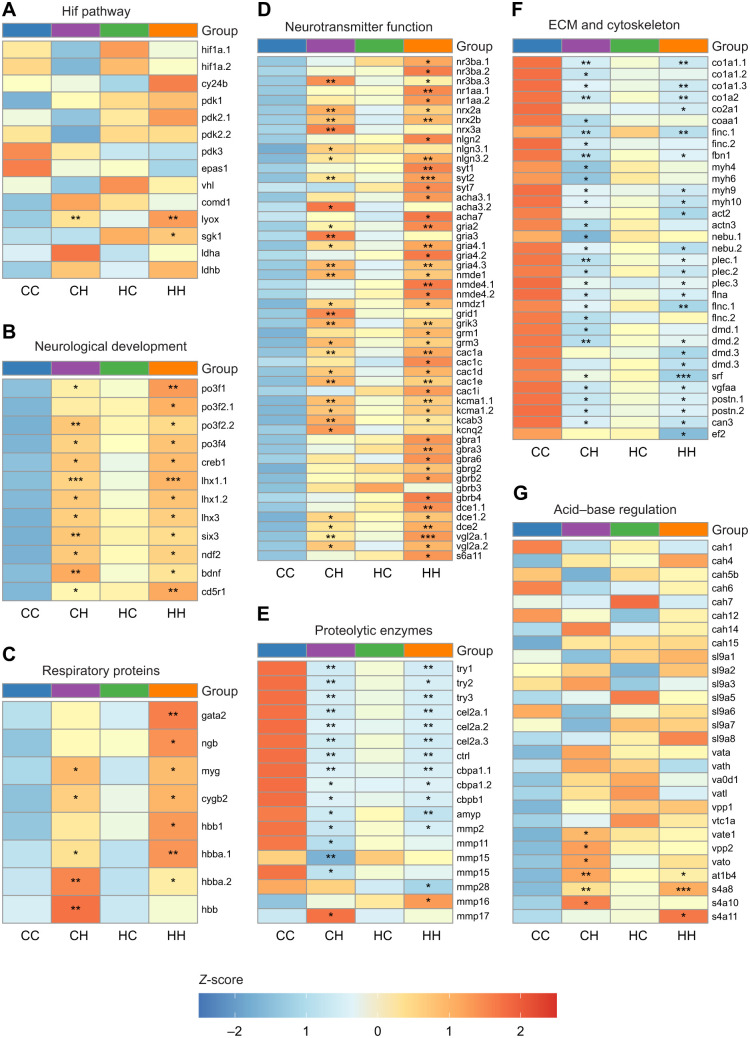
**Heatmaps of gene expression for selected cellular pathways.** Heatmaps showing gene expression (log_2_CPM normalized *Z*-score) across the four treatment groups within relevant transcriptional pathways. Rows show individual transcripts that are labeled by gene symbol on the right (‘.1’, ‘.2’, ‘.3’ indicate multiple isoforms for a single gene symbol). Asterisks inside the cell indicate that expression in that group was significantly different from that of CC larvae, the overall control group (*FDR*<*0.05, **FDR*<*0.01, ***FDR*<*0.001). (A) Hif pathway; (B) neurological development; (C) neurotransmitter function; (D) extracellular matrix (ECM) and cytoskeleton; (E) proteolytic enzymes; (F) respiratory proteins; and (G) acid–base regulation. For more details, see Discussion.

Both offspring groups exposed to HypOA exhibited substantial transcriptional changes compared with the overall control group. While the total number of DETs was similar between CH and HH larvae, most of the specific DETs differed between groups. Consequently, many enriched GO terms also varied; however, the most highly enriched processes remained similar. This suggests that parental environment had a limited impact on the overall transcriptional response to HypOA, despite some minor differences. For example, protein-lysine 6-oxidase was upregulated in both groups (Fig. 7A), a marker of hypoxia-induced factor (Hif) pathway activation, which regulates many cellular responses to low oxygen (Lin et al., 2020; Mandic et al., 2021). The expression of Hif proteins was not significantly affected by treatment conditions (Fig. 7A), but we identified several GO terms that function downstream of Hif activation, including nervous system development (Bonkowsky and Son, 2018), axonal pathfinding (Pocock and Hobert, 2008), neurotransmitter regulation (Paulding et al., 2002) and extracellular matrix homeostasis (Gilkes et al., 2014). The observed transcriptional responses may also reflect the smaller size of larvae reared under HypOA, which were at a slightly earlier developmental stage at the time of sampling. Additional experiments that allow HypOA-treated larvae to reach comparable developmental size would help resolve this uncertainty.

### Transcriptional effects associated with neurological development

The developing central nervous system is highly vulnerable to hypoxia, as insufficient oxygen levels impair critical developmental processes related to neurogenesis, axon pathfinding and synapse formation (Bonkowsky and Son, 2018). Reduced aerobic energy production and a loss of ion regulatory capacity leads to increased rates of neuronal cell death via apoptosis or necrosis (Banasiak et al., 2000). For example, zebrafish larvae exposed to a DO level of 10% a.s. displayed decreased neural proliferation in the forebrain and a downregulation of genes associated with the development of neurons, glia and oligodendrocytes (Mikloska et al., 2022). However, in this study, we observed an upregulation of genes associated with nervous system development, neurotransmitter activity and regulation of neuron membrane potential (Fig. 7B,D). This difference may be attributed to the more moderate hypoxia treatment (40% a.s.) applied here. However, it may have resulted from the simultaneous exposure to elevated *P*_CO_2__, which alters the expression of genes involved in neurological development and plasticity in several fish species (Costa et al., 2022; Lai et al., 2017; Suresh et al., 2023). We should note that the application of a combined HypOA treatment was to enable a feasible cross-generational design, but the transcriptional effects of combined HypOA remain poorly understood. Further research is needed to better understand how the combined effects of low DO and elevated *P*_CO_2__ affect neurodevelopment in aquatic animals, as the combined stressor elicits a unique transcriptional response compared with individual exposures (Cline et al., 2020; Iguchi et al., 2024).

Other upregulated genes in these pathways included several genes identified in our frontloading analysis, such as key transcription factors involved in nervous system development, including POU-domain class 3 transcription factors (Latchman, 1999), cyclic AMP-responsive element-binding protein 1 (Lee et al., 2018), LIM/homeobox proteins (Hobert and Westphal, 2000), homeobox protein six3 (Chao et al., 2010), neurogenic differentiation factors (Olson et al., 2001) and brain-derived neurotrophic factor protein (Anand and Mondal, 2020) (Fig. 7B). Also upregulated was cyclin-dependent kinase 5 activator 1 (also known as *p35*), a protein with anti-apoptotic effects that regulates neurogenesis and development of the retina in zebrafish (Leung et al., 2008) along with the neuroprotective protein cytochrome *c* oxidase subunit 5A (Tarasenko et al., 2017). This gene was upregulated in the brains of juvenile spiny damselfish (*Acanthochromis polyacanthus*) that were acutely exposed to elevated *P*_CO_2__ (Tsang et al., 2020).

Like other components of the central nervous system, the development of the retina is sensitive to hypoxia, and even modest reductions in available oxygen can impact visual performance (McCormick and Levin, 2017). Hence, smaller eyes may be an expected phenotypic response of larvae reared in oxygen-limiting conditions. However, we found that silverside larvae that were directly exposed to HypOA maintained an eye size relative to their body length that was comparable to that of larvae in the overall control group. This suggests that silverside offspring can make the necessary transcriptional adjustments to support eye growth in response to HypOA. Additionally, larvae from HypOA-treated parents but reared under control conditions exhibited larger eyes compared with all other groups, representing the only clear phenotypic difference observed between offspring from control and HypOA-treated parents. We hypothesize that modest transcriptional frontloading of genes involved in neurological and ocular development may have contributed to increased eye growth in the absence of the direct suppressive effects of HypOA. Further work is needed to understand the transcriptional drivers of this effect, and whether these changes have a functional effect on the visual sense of developing larvae.

### Within- and cross-generational effects of HypOA on neurotransmitter function

Larvae reared under HypOA exhibited extensive upregulation of genes associated with synapse formation and neurotransmitter regulation. This involved increased expression of several neurexin and neuroligin isoforms (Fig. 7D), proteins that form the trans-synaptic complexes between presynaptic and postsynaptic neurons and facilitate synapse formation and neurotransmitter release (Bottos et al., 2011). We also found an upregulation of several synaptotagmin isoforms (Fig. 7D), which are trafficking proteins located in the presynaptic membrane that regulate rapid neurotransmitter release (Chapman, 2008). Furthermore, exposed larvae showed increased expression of genes involved in excitatory neurotransmitter signaling, including acetylcholine receptors, and other ionotropic and metabotropic glutamate receptors (Fig. 7D). This pattern was accompanied by the upregulation of mRNAs encoding voltage-dependent calcium ion channels and voltage-gated potassium ion rectifier channels (Fig. 7D). Excitatory neurotransmission is vital for neuronal development and memory formation; however, secreted glutamate becomes excitotoxic under hypoxic conditions (Nilsson, 1995). Anoxia-tolerant species such as crucian carp (*Carassius carassius*) and goldfish (*Carassius auratus*) can survive extreme low oxygen, in part, through depression of neurological activity through increased production of GABA, the major inhibitory neurotransmitter in fish brains. GABA secretion alleviates neuronal excitotoxicity by inhibiting neurotransmission and promoting cell survival through anti-apoptotic and anti-oxidative effects (Nilsson, 1995). Interestingly, genes involved in GABA neurotransmission were identified as frontloaded in our exploratory analysis and expressed to a greater extent in larvae from HypOA-treated parents, including genes encoding GABA_A_ receptors, GABA_B_ receptors and proteins involved in GABA synthesis and transport (Fig. 7D).

The cross-generational effects on GABA expression and function are an important area for further investigation given its role in hypoxia tolerance and mediating behavioral dysfunction in acidified conditions (Schunter et al., 2018). In fish exposed to elevated *P*_CO_2__, acid–base regulatory adjustments alter ion gradients across neuronal membranes and cause a reversal of function in some GABA_A_ receptors, resulting in excitatory instead of inhibitory signaling (Heuer et al., 2019; Nilsson et al., 2012). Interestingly, parental exposure to elevated *P*_CO_2__ has been shown to offset behavioral effects in offspring, suggesting that cross-generational changes to transcriptional flexibility can restore normal GABA function (Schunter et al., 2016). Our findings offer further support that cross-generational effects can alter expression of neurotransmitters and associated proteins.

### HypOA induced metabolic depression

Metabolic depression is a primary strategy employed by fish to survive periods of low DO (Richards, 2009). For example, the anoxia-tolerant embryos of the annual killifish (*Austrofundulus limnaeus*) reduce metabolic rate in an oxygen-dependent manner and do not increase ATP production through anaerobic metabolism (Anderson and Podrabsky, 2014). In this study, Atlantic silverside larvae responded in a similar manner through delayed development, reduced size at hatch and slower rates of post-hatch larval growth. Furthermore, transcripts encoding proteins required for anaerobic energy production (e.g. lactate dehydrogenases) were not significantly affected by parental or offspring treatment (Fig. 7A). We also found a downregulation of genes involved in somatic growth and tissue development. These included major components of the extracellular matrix, such as collagens, fibronectin and fibrillin, as well as cytoskeleton components and other proteins involved in muscle development and contraction, including myosin heavy chains, actins, nebulins, plectins, filamens and dystrophins (Fig. 7F). This pattern was accompanied by a downregulation of growth factors and proteins implicated in tissue remodeling and muscle development, including serum response factor (Miano et al., 2007), periostin (Kudo et al., 2004) and calpain-3 (Prykhozhij et al., 2023) (Fig. 7F).

In part, this transcriptional pattern may reflect that larvae reared under HypOA were smaller and less developed than control larvae at the time of sampling. However, the fact that the suppressive pattern of these genes appeared to be frontloaded in larvae from HypOA-treated parents suggests an active response to HypOA, allowing for metabolic savings by reducing the cellular machinery needed to support muscle development and movement, as well as a general reduction in the synthesis of these highly abundant proteins (Richards, 2009). Similar adaptive responses to hypoxia related to muscle contraction proteins, extracellular matrix components and cytoskeletal proteins have been observed in long jawed mudsucker (*Gillichthys mirabilis*) and zebrafish (Gracey et al., 2001; Ton et al., 2003). Whether these transcriptional changes associated with direct or parental exposure to HypOA impact muscle integrity, swimming performance and wound healing in developing offspring carries important implications for future research.

### Expression of respiratory and acid–base regulatory proteins

Fish exposed to hypoxia typically upregulate globin proteins to enhance oxygen transport (Richards, 2009) and acidification may similarly induce their expression as a result of their role in the oxidative stress response (Tiedke et al., 2013). In Atlantic silverside larvae reared under HypOA, we observed strong upregulation of globin-related mRNAs, including myoglobin, cytoglobin-2 and hemoglobin subunit beta-1, with the latter being the most highly expressed hemoglobin transcript (>40-fold increase; Fig. 7C). Neuroglobin, which protects neurons from ischemic injury (Fuchs et al., 2004), was also identified as potentially frontloaded in larvae from HypOA-treated parents, and was only significantly upregulated in HH larvae and not in the CH group when compared with the overall control group. This coincided with increased gata2 transcription, a regulator of hematopoietic stem cell differentiation (Gioacchino et al., 2021). A similar transgenerational response has been observed in zebrafish, where paternal hypoxia exposure enhanced offspring hemoglobin expression and hypoxic tolerance (Ragsdale et al., 2022).

While hypoxia and acidification can elevate acid–base regulatory demands in fish (Tseng et al., 2013), HypOA-exposed silverside larvae did not show a consistent upregulation of genes involved in acid–base balance (Fig. 7G). Carbonic anhydrases, which mediate CO_2_ excretion and pH regulation (Gilmour et al., 2009), and membrane ion transporters such as sodium/hydrogen exchangers and V-type ATPases, were largely unaffected (Fig. 7G). Notably, sodium/potassium ATPases were downregulated, though some V-type ATPase subunits were slightly upregulated in CH larvae only (Fig. 7G). These findings indicate that Atlantic silverside larvae possess sufficient basal acid–base regulatory capacity to withstand acidification without major transcriptional adjustments (Kwan et al., 2021). However, because ion regulation is energy intensive and hypoxia necessitates metabolic depression (Perry and Gilmour, 2006; Richards, 2009), simultaneous exposure to low DO may increase the risk of acidosis, potentially exacerbating negative interactive effects observed in silverside offspring (DePasquale et al., 2015; Miller et al., 2016; Schwemmer et al., 2020).

### Conclusion

By applying a cross-generational factorial design, we showed that parental exposure to nocturnal HypOA conditions prior to spawning did not increase offspring tolerance to chronic HypOA during embryo and larval development. RNAseq analysis revealed that silverside offspring mounted a robust transcriptional response to HypOA, characterized by the upregulation of genes involved in neural development, synaptic plasticity and ion regulation. This pattern suggests prioritization of the development and protection of the central nervous system, potentially at the expense of somatic growth, as indicated by the downregulation of genes associated with extracellular matrix organization and muscle development. However, we cannot rule out the possibility that this pattern was partially influenced by slight developmental delays in HypOA-reared larvae.

Parental HypOA exposure subtly influenced the transcriptional plasticity of their offspring, resulting in reduced gene expression differences between control and HypOA-reared larvae within the HypOA-treated parental group. This effect was accompanied by modest evidence of transcriptional frontloading in a subset of stress-response genes, although these changes were not statistically significant at the individual gene level. While such cross-generational effects may help prepare offspring for recurring environmental stressors such as HypOA, they did not confer measurable phenotypic benefits under our experimental conditions. Future research should investigate the functional consequences of frontloading on traits relevant to HypOA tolerance, including potential trade-offs affecting behavior, oxygen acquisition and transport, metabolic rate and long-term growth and development. Additional studies are also needed to uncover the maternal and epigenetic mechanisms underlying cross-generational plasticity and to assess how the timing, intensity and variability of parental exposure shape offspring responses in dynamic coastal environments.

## Supplementary Material

10.1242/jexbio.249726_sup1 Supplementary information

Dataset 1. Sheet 1: all_DEG_comparisons. This sheet presents the edgeR output for all six pairwise differential expression comparisons. Each row represents a gene, with columns detailing the comparison group number (*pairwise_group*), the specific treatment groups being compared (Comparison), and the gene identification code based on *Menidia menidia* annotation (menidiaID). Additional columns include the abbreviated gene name (*geneID*), average expression level in log counts per million (*logCPM*), log fold change for the given comparison (logFC), false discovery rate (FDR), and associated Gene Ontology (GO) terms (*GOTerms*). Sheet 2: GO_reductionAnalysis_results. Results of GO term redundancy reduction analysis using the rrvgo package. Each row represents an individual GO term after redundancy filtering. Columns include the identifier for the GO analysis group, representing each pairwise comparison (*GO_AnalysisGroup*), the corresponding semantic similarity root node (*root_node*), and information on the representative parent term including its GO ID (*parentID*), description (*parentTerm*), and parent term enrichment score (*parentEnrichScore*). Redundancy metrics include the uniqueness of the term overall (*termUniqueness*), within its cluster (*termUniquenessWithinCluster*), and its dispensability score (*termDispensability*). The original (*non-reduced*) GO term is also reported, with its ID (*individualTermID*) and description (*individualTermDescription*), followed by enrichment statistics: gene ratio (*GeneRatio*), background ratio (*BgRatio*), raw p-value (*pvalue*), adjusted p-value (*p.adjust*), and q-value (*qvalue*). The final column (*enrichingGenes*) lists genes contributing to the enrichment of each individual GO term.

## Appendix

### Methodology for assessing frontloaded transcripts

To explore the degree of transcriptional frontloading in larvae from HypOA-treated parents, we analyzed gene expression patterns using methods adapted from Barshis et al. (2013). We extracted transcript IDs for all DETs identified in the control–HypOA (CH) versus control–control (CC) pairwise comparison (*N*=1603) as the core set of genes responding to direct HypOA exposure in offspring. For each transcript ID, we extracted normalized and variance-stabilized counts per million (CPM) as well as log fold-change (log_2_FC) values from the CH versus CC and HypOA–HypOA (HH) versus HypOA–control (HC) pairwise comparisons. Next, we calculated a constitutive expression ratio (CER) to estimate how parental environmental history influenced gene expression in larvae reared under control conditions. The CER was calculated as the ratio of gene expression (CPM) in HC larvae relative to CC larvae:

(rmA1)

The standard deviation (s.d.) for this ratio was computed using the error propagation equation for ratio data:

(rmA2)

Next, we calculated the raw FC values separately for upregulated (FC=2^log_2_FC^) and downregulated DETs (FC=0.5^log_2_FC^). FCs were used to calculate the fold-change ratio (FCR) for each DET to quantify how parental acclimation influenced gene expression plasticity between offspring treatments:

(rmA3)

*edgeR* does not provide variance estimates for log_2_FC values, so we could not propagate error for the FCR in the same way as for the CER. Both ratios were log-transformed to improve visualization in a scatterplot. Genes were categorized as frontloaded if they met all of the following criteria: (1) the CER was positive for upregulated DETs and negative for downregulated DETs, (2) the ±s.d. of the CER did not include zero, and (3) the FCR was less than 0.9, indicating that the fold-change was at least 10% smaller in the HH versus HC comparison relative to the CH versus CC comparison (used as a proxy as variance estimates for FCR were unavailable). GO enrichment analysis of frontloaded DETs was conducted using the methods described above.

### Results of frontloading analysis

We identified 376 upregulated and 364 downregulated DETs that met the criteria for transcriptional frontloading: (1) constitutive expression changes in HC larvae that matched the directional response of larvae directly exposed to HypOA, (2) the pattern was consistent across individual HC larvae (i.e. s.d. for CER did not intersect 0) and (3) the effect led to at least a 10% reduction in logFC for the HH versus HC pairwise comparison compared with CH versus CC comparison (Fig. A1). In general, the FCR became more negative as the absolute value of the CER increased (Fig. A1), indicating that expression changes in HC larvae were driving the reduced transcriptional plasticity observed in larvae from HypOA-treated parents. Frontloaded DETs were enriched in many of the same top GO BP and MF terms that were enriched in the DETs exhibited by CH and HH larvae (Fig. A2).
