# Supplementary material for: Cross-generational plasticity in Atlantic silversides (Menidia menidia) under the combined effects of hypoxia and acidification
Source: J Exp Biol. 2025 Jul 25;228(15):jeb249726. doi: 10.1242/jeb.249726 (PMC12319412; doi:10.1242/jeb.249726)
Supplement: Supplementary information [file jexbio-228-249726-s1.pdf]

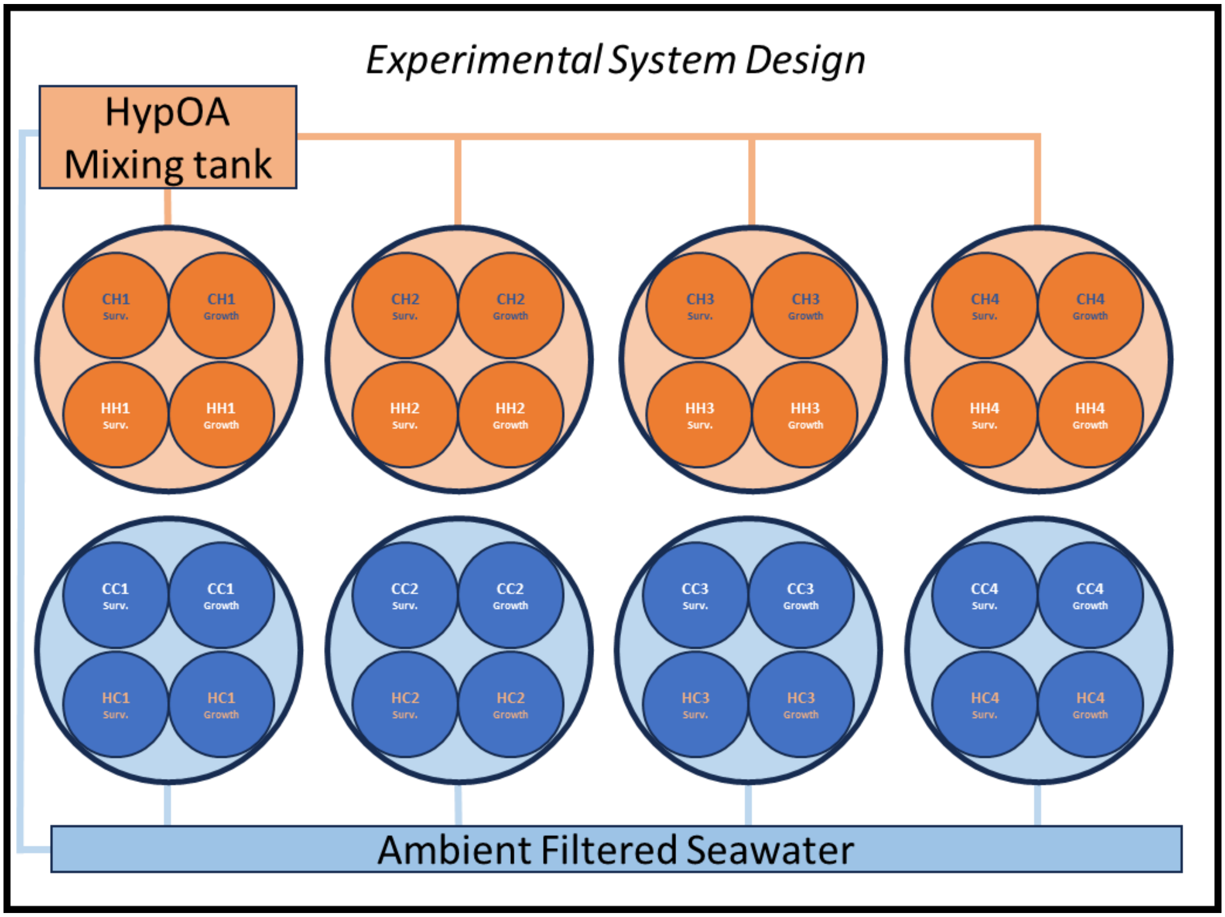

Fig. S1. Schematic of experimental design during offspring exposures. The setup was composed of eight 900-L circle tanks that housed fish and one 600-L mixing tank. Four circle tanks received ambient laboratory seawater (21°) set to control treatment conditions [dissolved oxygen (DO):100% air saturation (a.s.) / pCO_2: 650 µatm]. The other four circle tanks received HypOA-adjusted seawater [DO: 40% a.s. / pCO_2: 2300 µatm]. HypOA conditions were maintained in the mixing tank using a computer-controlled CO_2 and N_2 dosing system and then pumped to induvial circle tanks. After fertilization, each tank received embryos from both parental acclimation groups to form four offspring treatment groups: control parents × control offspring (CC); control parents × HypOA offspring (CH); HypOA parents × control offspring (HC); and HypOA parents × HypOA offspring (HH). After fertilization, each tank was equipped with four 20-L rearing containers: one container for each parental treatment to measure survival, and another to serve as the source for subsamples for growth and transcriptomic traits. Note that main tanks could hold up to eight containers at a time, which facilitated the transfer of hatched larvae to a new rearing container during the multi-day hatching period.

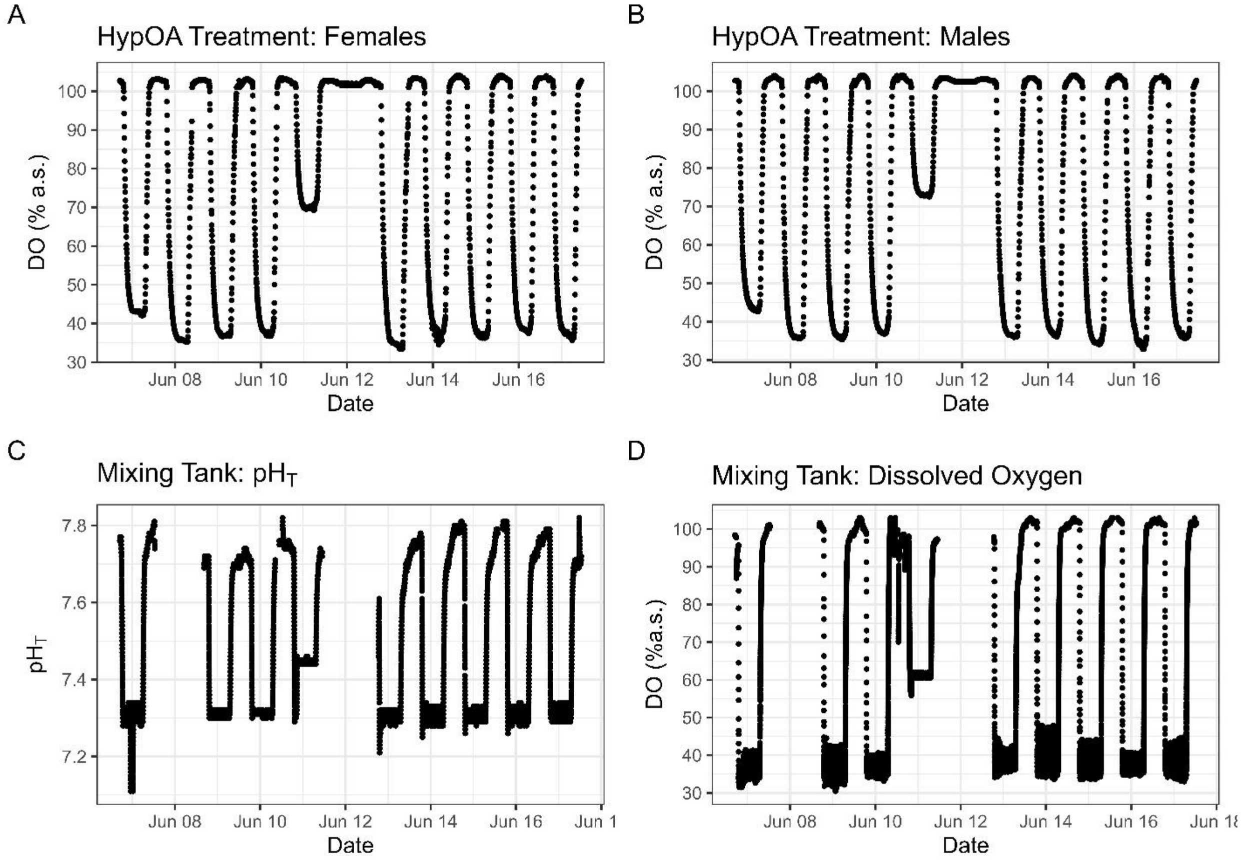

Fig. S2. Parental conditions tanks for females (A) and males (B) during the eleven-day treatment period to HypOA. Dissolved oxygen was recorded every 5 min using HOBO Dissolved Oxygen Data Loggers (model: U26-001). The loggers were calibrated daily with a two-point calibration following the manufacturer's guidelines. Mixing tank conditions for pH (C) and dissolved oxygen (D) during the eleven-day parental treatment period to HypOA. A gap in mixing tank data on June 7-8 was the result of a data recording error while the system continued to function normally. However, a software malfunction occurred during the nights of June 11 and June 12 such that HypOA conditions were not fully achieved in parent tanks during those two nights.

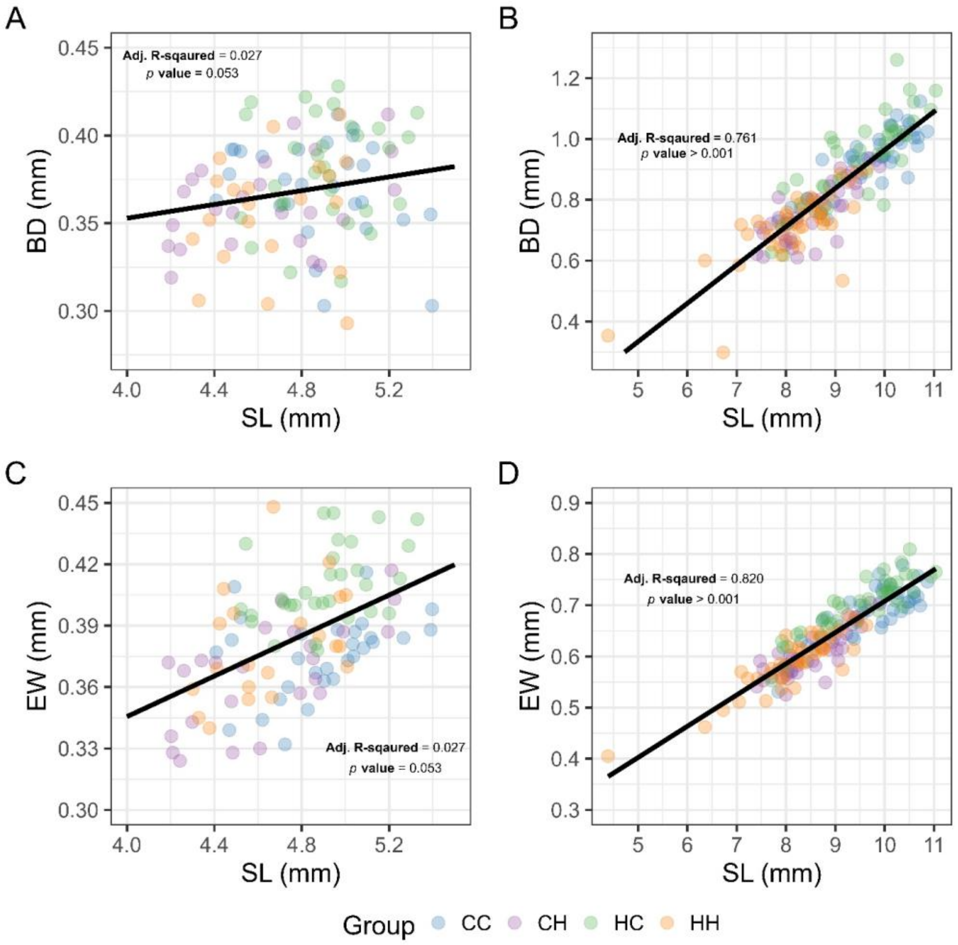

Fig. S3. Linear regression fits for standard length to body depth and standard length to eye width on pooled data from all treatment groups. Panels A and C show data from hatchlings and B and D show data from larvae sampled at 19 days post fertilization. Colored circles represent individual larvae from different treatment groups. The adjusted r-squared and significance level (*p* value) is shown for each regression fit.

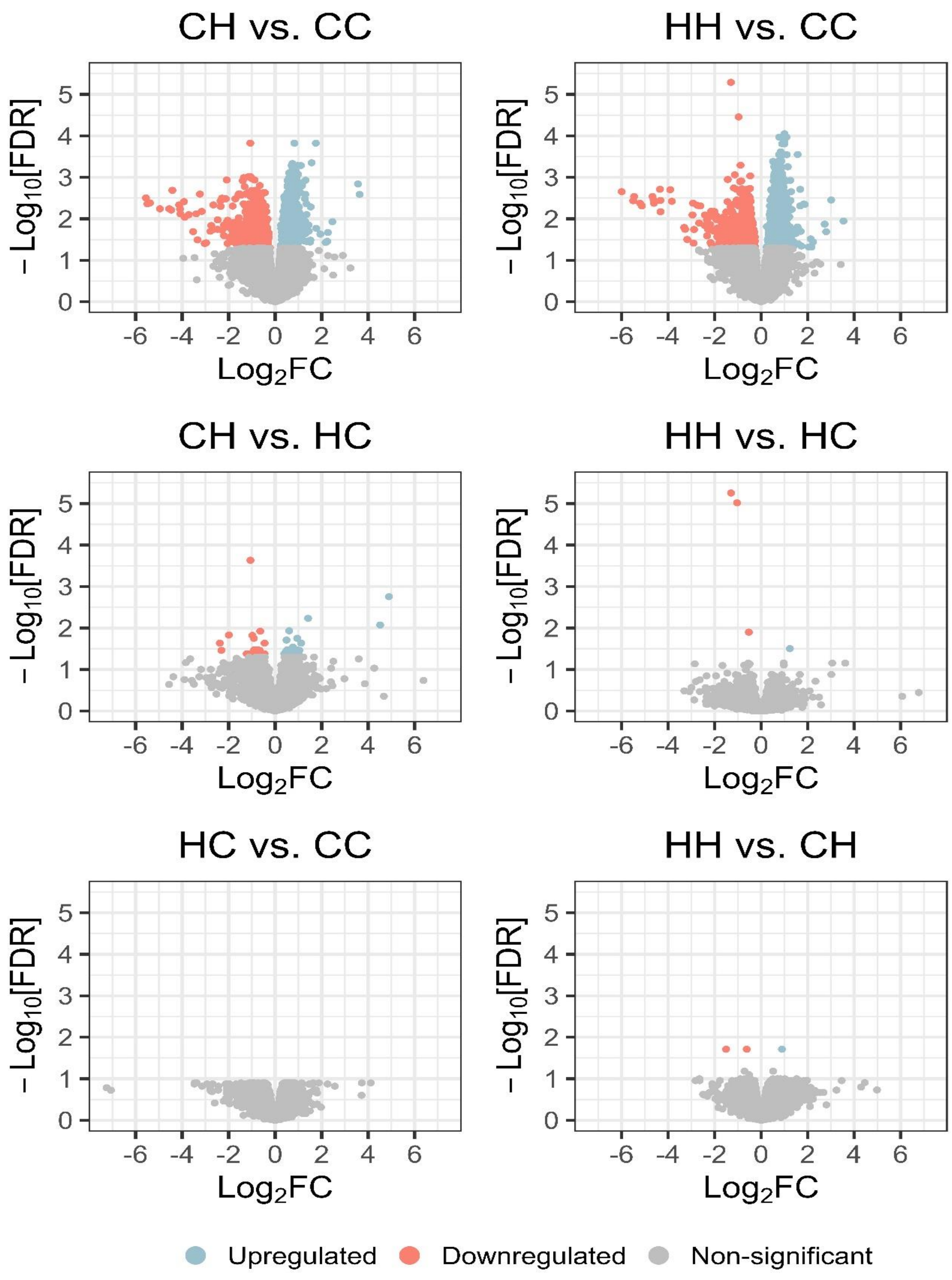

Fig. S4. Volcano plots showing log fold change (log2FC) and significance level (-log10[FDR]) for all transcripts all pairwise treatment comparison. Each transcript is shown as an individual circle and are colored by their response: significantly upregulated (blue), significantly downregulated (red), or not significant (grey).

Journal of Experimental Biology • Supplementary information

Table S1. Adult silversides that were conditioned to either control and HypOA conditions and used to fertilize experimental embryos. The number of female and male silversides used in the fertilization is noted in parentheses in the sex column, as well as average (±s.d.) total length (TL, cm) and wet weight (wW, g).

Parental Treatment	Sex	TL (cm)	wW (g)
Control	Female (7)	11.0±0.8	6.2±1.3
	Male (17)	8.7±1.1	3.7±1.2
HypOA	Female (7)	8.8±0.6	3.6±0.6
	Male (20)	9.2±1.1	4.3±1.7

Dataset 1. Sheet 1: all_DEG_comparisons. This sheet presents the edgeR output for all six pairwise differential expression comparisons. Each row represents a gene, with columns detailing the comparison group number (*pairwise_group*), the specific treatment groups being compared (Comparison), and the gene identification code based on *Menidia menidia* annotation (*menidiaID*). Additional columns include the abbreviated gene name (*geneID*), average expression level in log counts per million (*logCPM*), log fold change for the given comparison (logFC), false discovery rate (FDR), and associated Gene Ontology (GO) terms (*GOTerms*). Sheet 2: GO_reductionAnalysis_results. Results of GO term redundancy reduction analysis using the rrvgo package. Each row represents an individual GO term after redundancy filtering. Columns include the identifier for the GO analysis group, representing each pairwise comparison (*GO_AnalysisGroup*), the corresponding semantic similarity root node (*root_node*), and information on the representative parent term including its GO ID (*parentID*), description (*parentTerm*), and parent term enrichment score (*parentEnrichScore*). Redundancy metrics include the uniqueness of the term overall (*termUniqueness*), within its cluster (*termUniquenessWithinCluster*), and its dispensability score (*termDispensability*). The original (non-reduced) GO term is also reported, with its ID (*individualTermID*) and description (*individualTermDescription*), followed by enrichment statistics: gene ratio (*GeneRatio*), background ratio (*BgRatio*), raw p-value (*pvalue*), adjusted p-value (*p.adjust*), and q-value (*qvalue*). The final column (*enrichingGenes*) lists genes contributing to the enrichment of each individual GO term.

Available for download at
https://journals.biologists.com/jeb/article-lookup/doi/10.1242/jeb.249726#supplementary-data

Journal of Experimental Biology • Supplementary information
